# Contrast of Backscattered Electron SEM Images of Nanoparticles on Substrates with Complex Structure

**DOI:** 10.1155/2017/4907457

**Published:** 2017-04-06

**Authors:** Thomas Kowoll, Erich Müller, Susanne Fritsch-Decker, Simon Hettler, Heike Störmer, Carsten Weiss, Dagmar Gerthsen

**Affiliations:** ^1^Laboratory for Electron Microscopy, Karlsruhe Institute of Technology (KIT), Campus South, Engesserstr. 7, 76131 Karlsruhe, Germany; ^2^Institute of Toxicology and Genetics, Karlsruhe Institute of Technology (KIT), Campus North, Hermann-von-Helmholtz-Platz 1, 76344 Eggenstein-Leopoldshafen, Germany

## Abstract

This study is concerned with backscattered electron scanning electron microscopy (BSE SEM) contrast of complex nanoscaled samples which consist of SiO_2_ nanoparticles (NPs) deposited on indium-tin-oxide covered bulk SiO_2_ and glassy carbon substrates. BSE SEM contrast of NPs is studied as function of the primary electron energy and working distance. Contrast inversions are observed which prevent intuitive interpretation of NP contrast in terms of material contrast. Experimental data is quantitatively compared with Monte-Carlo- (MC-) simulations. Quantitative agreement between experimental data and MC-simulations is obtained if the transmission characteristics of the annular semiconductor detector are taken into account. MC-simulations facilitate the understanding of NP contrast inversions and are helpful to derive conditions for optimum material and topography contrast.

## 1. Introduction

Scanning electron microscopy (SEM) is a widely applied characterization technique to study the properties of nanoparticles (NPs). Secondary electron (SE) SEM images provide information on surface topography, size, and size distribution of NPs. The atomic number sensitivity of backscattered electron (BSE) SEM images can be exploited to distinguish NPs with different chemical composition to reveal, for example, contamination particles. BSEs are by definition electrons with a kinetic energy above 50 eV. However, a significant BSE fraction is elastically scattered and a maximum of the energy distribution of BSEs exists close to the primary electron (PE) energy *E*_0_ [1]. BSE SEM images depend on the backscattered electron coefficient *η* which is defined by the number of BSEs per primary electron. A considerable amount of work has been devoted to measurements and calculations of the atomic number dependence of the BSE coefficient. Early work on BSE SEM imaging was performed by Niedrig [1] and Joy [2, 3], and a summary is given by Reimer [4]. There has been recently a strong tendency towards lowering *E*_0_ which was initiated by the improvement of the electron optics and electron sources. Lower *E*_0_ values reduce the size of the interaction volume and lead to a substantial improvement of the resolution. This applies in particular for images with BSEs with exit depths that are large compared to secondary electrons. Along this line, Cazaux [5] summarized measured and calculated *η* values for a wide range of elements at electron energies below 5 keV. Overall considerable knowledge is available on *η* and its dependence on the atomic number and orientation of the sample surface with respect to the incident beam and *E*_0_.

Despite that, BSE SEM imaging has been rarely exploited to quantify information from BSE images. This must be attributed to the fact that the properties of the detection system as well as brightness and contrast settings need to be properly controlled and taken into account for the image evaluation. One of the few examples was presented by Sánchez et al. [6] who determined the average atomic number *Z* of a set of polished metal and mineral samples based on the measured BSE intensity. Instrumental effects were taken into account by using reference samples with known *Z*, and samples with unknown *Z* were studied without changing the imaging parameters.

Topography effects can be excluded in BSE images if polished surfaces are analysed. This cannot a priori be assumed for nanoscaled objects with a pronounced topography like NPs. Moreover, the influence of the substrate on NP contrast cannot be neglected. A study of gold NPs imaged by BSEs was published by Hirsch et al. [7]. They investigated BSE SEM contrast of Au NPs with sizes between 2 nm and 40 nm on a silicon substrate. Monte-Carlo- (MC-) simulations of BSE contrast revealed pronounced dependence of the NP contrast on *E*_0_. A contrast maximum occurs if the NP diameter corresponds to the electron range in gold, which requires the adaption of *E*_0_ to the NP size. The NP contrast decreases and even vanishes if *E*_0_ is significantly increased beyond the optimum *E*_0_. The BSE contrast of NPs on bulk substrates will be also affected by changes of *η* with decreasing *E*_0_ [5, 7]. Overall, unexpected effects can be foreseen for BSE SEM images of NPs on bulk substrates. 

In our study we focus on the contrast of SiO_2_ NPs on glass and glassy carbon substrates with an indium-tin-oxide (ITO) coating. This type of substrate is interesting for correlative SEM and light microscopy studies of biological samples as demonstrated by Pluk et al. [8]. We aim towards a quantitative understanding of BSE contrast as a function of the working distance and PE energy to optimize NP contrast. MC-simulations are employed to simulate the NP contrast on the ITO/glass and ITO/carbon substrates for comparison with experimental data to understand contrast formation. We will show that the properties of the used annular semiconductor detector need to be taken into account for quantitative comparisons of simulated and experimental data. This concerns the limited detection angle range and decreasing detector efficiency for low-energy electrons. Substantial deviations from the expected *Z*-contrast and even contrast inversions occur due to the small NP size, topography effects, and the complex substrate structure with a high *Z* surface coating in combination with small *E*_0_ values. Finally, we will provide a generally applicable strategy on how BSE contrast of nanoscaled objects on complex bulk substrates can be optimized.

## 2. Materials and Methods

### 2.1. Sample Preparation

The investigated samples are a product of studies on SiO_2_ NP uptake in A549 cancerogenous human lung epithelial cells. In these experiments cells, cultured as previously described by Panas et al. [9], were seeded onto ITO-coated substrates and incubated with nonporous amorphous SiO_2_ NPs (Postnova Analytics, Landsberg am Lech, Germany) with a diameter of 90 ± 8 nm (nominal diameter 100 nm according to manufacturer information). SiO_2_ NPs were deposited directly on the ITO-coated substrates between the cells. These regions are studied in the present work. The substrates were contacted with conductive silver on aluminium sample holders (Plano GmbH, Wetzlar, Germany) with a diameter of 32 mm for the SEM investigations.

Two different substrate types were used. Type 1 consists of 160 ± 5 nm thick ITO layers on glass (amorphous SiO_2_) denoted by* ITO160 *in the following. For comparison, glassy carbon substrates covered by 22 ± 5 nm thin ITO layers were used denoted by* ITO22*. The second substrate type was chosen because glassy carbon is characterized by a low *η* value compared to the SiO_2_ substrate. It will later become obvious that the thin ITO layer on the glassy carbon substrate will considerably contribute to the understanding of BSE image formation. The* ITO160 *substrates were purchased from PGO (Iserlohn, Germany), while the* ITO22 *ones were manufactured in-house by electron-beam deposition (Kurt J. Lesker Company, Hastings, UK) from ITO pieces on glassy carbon substrates (HTW, Thierhaupten, Germany).

### 2.2. Transmission Electron Microscopy

Transmission electron microscopy (TEM) and scanning transmission electron microscopy (STEM) combined with energy dispersive X-ray spectroscopy (EDXS) were performed to determine the thickness and composition of the ITO layers. (S)TEM was performed with a FEI Osiris ChemiSTEM operated at 200 kV and equipped with four Bruker silicon drift detectors. TEM cross-section specimens were prepared by focused ion-beam (FIB) milling. The measured compositions are included in Table 1. We note that substantial discrepancies between experimental and simulated BSE contrast of NPs result if the nominal instead of the real compositions are used in the MC-simulations.

**Figure 1:**
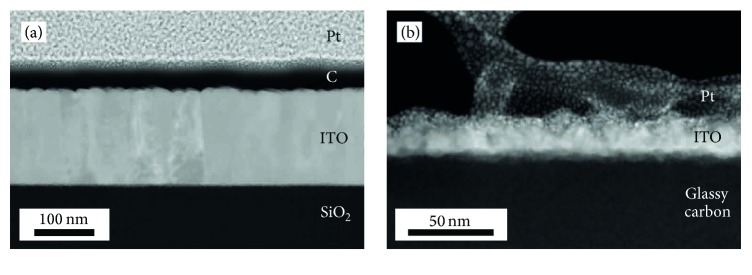
200 keV cross-sectional HAADF STEM images of (a) ITO160 and (b) ITO22 covered by a protective Pt-layer.

Figure 1 shows high-angle annular dark-field (HAADF) STEM images of* ITO160* (Figure 1(a)) and* ITO22* (Figure 1(b)) in cross-section perspective. The ITO layers show bright contrast compared to the glassy carbon and SiO_2_ substrates. The samples are covered by Pt-layers for protection during FIB milling. The dark layer between* ITO160* and the platinum layer in Figure 1(a) is an additional carbon layer, which was necessary to improve electrical conductivity for other experiments and has no further relevance for this work. The ITO layer in* ITO160* appears dense with a homogeneous contrast and exhibits thickness variations of ±5 nm. The inhomogeneous contrast of the ITO layer in* ITO22* suggests that some porosity is present and indicates that the average ITO density is smaller than the nominal one. MC-simulations performed with a reduced density of 5.0 g/cm^3^ instead of 7.1 g/cm^3^ for* ITO22* indeed agree well with the experimental results.

### 2.3. Scanning Electron Microscopy

All samples were investigated in a FEI Quanta 650 ESEM, equipped with a Schottky field emission gun and an annular silicon solid-state BSE detector with an active detector area of approximately 200 mm^2^. The specimen stage was untilted and images were taken at normal incidence. SE images were acquired with an Everhart-Thornley detector. Three series of BSE images were acquired for* ITO160* and* ITO22* by varying *E*_0_ between 3 and 17 keV while keeping the working distance (WD) constant at 4 mm, 6 mm, and 10 mm. Moreover, two series were acquired with different WD between 4 and 12 mm at constant *E*_0_ of 5 keV and 10 keV. Every image was taken at different, but adjacent specimen areas to minimize contamination artefacts.

Images with 2048 × 1768 pixels and 1.46 nm pixel size were taken corresponding to a magnification of ×100.000. Spot size 3, 10 *μ*s dwell time, and 16-bit greyscale resolution were chosen. Brightness and contrast values were adjusted to strictly avoid over- and undersaturation of the signal over a whole image series. For energies below 5 keV, the contrast had to be occasionally increased within one series to obtain reasonable signal intensities. The investigation of the influence of contrast and brightness variations on the measured NP contrast at *E*_0_ = 5 keV and WD = 4 mm showed that only brightness alters the NP contrast considerably, whereas varying contrast setting only leads to minor changes. For each detector setup, that is, brightness and contrast settings, images with a blanked beam were acquired to obtain black level intensities (see data analysis section below).

### 2.4. Data Analysis

The NP contrast is given by

(1) $$\begin{array}{c} C=\frac{I_{NP}-I_{sub}}{I_{sub}-I_{black}} \end{array}$$

with the NP image intensity I_NP_, the image intensity of the substrate I_sub_, and the black level intensity of the particular detector setup I_black_. The images were analysed with the software ImageJ [10]. To prevent errors in the subsequent averaging process, first potential bright and dark outliers (dark pixels and hotspots) were removed using the respective software feature. This filter replaces a pixel with the median intensity of the pixels in its surrounding if the pixel intensity differs by more than a certain threshold value from the median. The filter parameters were set as follows: pixels = 7 and threshold = 2000. Using the oval selection tool, a circle with a diameter between 40 nm and 50 nm was placed concentrically on a NP. The intensities of individual NPs I_NP_i__ were obtained by averaging the pixel intensities within the selected region. The substrate intensity I_sub_ was obtained by averaging the intensities of 10 large free areas at different positions. Finally, I_black_ was measured for each detector setup using images acquired with blanked beam. The contrast of each NP C_i_ was calculated on the basis of (1). Finally the average contrast $\overset{-}{\text{C}}$ was determined by averaging all C_i_. The resulting error represents the standard deviation of $\overset{-}{\text{C}}$ from an ensemble of 20 NPs. The evaluation of a relatively small NP ensemble is justified by the relatively small standard deviations of the NP contrast.

### 2.5. Monte-Carlo Simulations

MC-simulations were performed with the NISTMonte program [11] which was modified to take the detector properties into account. Two structural models were defined to calculate substrate and NP BSE intensities. Bulk substrates (amorphous SiO_2_ or glassy carbon) covered by an ITO layer with 160 or 22 nm thickness are assumed. For the calculation of the NP BSE intensity, a SiO_2_ NP with a diameter of 90 nm is placed on top of the substrate. Material parameters for NP and substrates are summarized in Table 1.

Simulation parameters were set as follows: 10^6^ trajectories, Gaussian beam with full width at half maximum of 1 nm, and PE energies *E*_0_ between 2 and 17 keV. We refrain from using PE energies below 2 keV, because the scattering cross-sections, especially Screened Rutherford cross-sections, fail to describe the backscattering coefficient *η* as surface barrier effects come into play [5]. Screened Rutherford (ScR) [12] and Czyzewski (Cz) Mott [13] scattering cross-sections were used and compared with respect to their validity to describe the experimental data.

One modification of NISTMonte is related to the operation principle of a semiconductor BSE detector. The detected BSE intensity in a semiconductor detector is determined by the number of electron-hole pairs which are generated by the BSEs. BSEs with different kinetic energies correspondingly generate different numbers of electron-hole pairs. Hence, the measured BSE signal cannot be directly attributed to the number of BSEs but depends also on the energy of each BSE. The calculation of the energy loss by the continuous slowing down approximation by Joy and Luo [14] is already implemented in NISTMonte. However, the energy of the individual BSEs is discarded in further processing. For the MC-simulations in this work, we sort BSEs into bins according to their scattering angle and monitor the BSE energy in addition to the BSE number. The number of electron-hole pairs generated in the semiconductor detector can be calculated by summing up the BSE energies in the bins for the corresponding scattering angle range. Since the greyscale value in a BSE image is proportional to the number of electron-hole pairs generated in the detector, the NP contrast is calculated by

(2) $$\begin{array}{c} C=\frac{E_{NP}-E_{sub}}{E_{sub}} \end{array}$$

with the overall BSE energy for the NPs on the substrate E_NP_ and for the mere substrate E_sub_.

Furthermore, a correction related to the efficiency of Si-detectors in converting electrons into electron-hole-pairs is included. The detector efficiency decreases with decreasing energy of the detected electrons due to the front metal coating of the detector. This can be described by a linearly decreasing transmission probability through the protective layer *T* = *E*_BSE_/E_th_ for BSEs with energies *E*_BSE_ starting from the threshold energy *E*_th_, which denotes the electron energy at which 100% transmission is achieved. For higher BSE energies the transmission is 100% and does not depend on *E*_BSE_ anymore.

**Figure 2:**
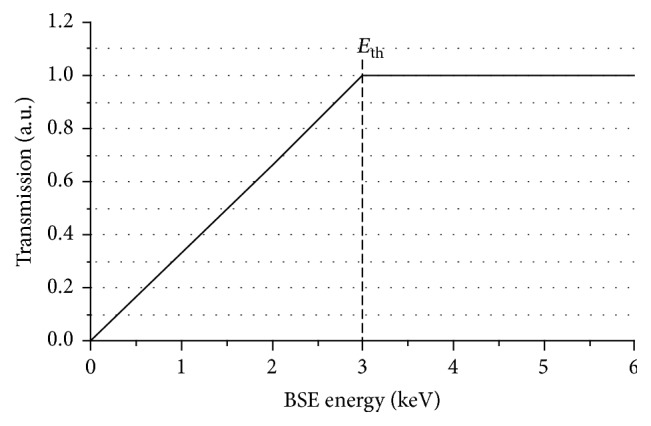
Scheme of BSE transmission for a semiconductor detector as a function of BSE energy.

Figure 2 shows the assumed transmission characteristic of the protective layer as a function of *E*_BSE_. *E*_th_ was set to 3 keV according to the information provided by the microscope manufacturer. The value of the threshold energy is important for low-energy BSE imaging because it determines an additional energy loss of the BSEs before they reach the detector. Hence, the energy of each electron has to be corrected before it is assigned to a bin taking into account the reduced transmission probability and the additional energy loss. This can be achieved by integrating the transmission curve shown in Figure 2 from 0 to *E*_BSE_, which yields the transmitted energy *E*_trans_ if *E*_BSE_ is below *E*_th_ (3). Equation (4) describes the energy correction for BSEs with energies above *E*_th_.

(3) Etrans=∫0EBSEEEthdE=EBSE22Ethfor  EBSE<Eth,

(4) Etrans=∫0EthEEthdE+∫EthEBSEdE=EBSE−12Ethfor  EBSE≥Eth.

The distance between detector and specimen determines the detected angular BSE distributions which need to be exactly known for the MC-simulations. The nominal WD settings of the microscope, however, denote the distance between specimen surface and pole piece and must be reduced by the BSE detector thickness of 2.15 mm. Hence, the nominal WDs were corrected in this work for the calculation of the minimum and maximum scattering angles. The smallest WD of 4 mm corresponds to an angular range of 1.78 rad–2.13 rad. It increases to 2.43 rad–2.85 rad for the largest WD = 12 mm. The scattering angle *θ* is defined as the angle with respect to the electron incidence direction. *θ* = 0 rad corresponds to forward scattering, while *θ* = 3.14 rad corresponds to backscattering perpendicular to the surface.

The errors for the MC-data were calculated according to Gaussian error propagation on the basis of uncertainties with respect to the ITO density of ±0.5 g/cm^3^, SiO_2_ density of ±0.2 g/cm^3^, NP diameter of ±8 nm, and ITO layer thickness of ±5 nm. Statistical errors can be neglected due to the large number (10^6^) of simulated electrons.

## 3. Experimental Results

The NP contrast was systematically investigated as a function of the working distance and primary electron energy for the two different substrates.

**Figure 3:**
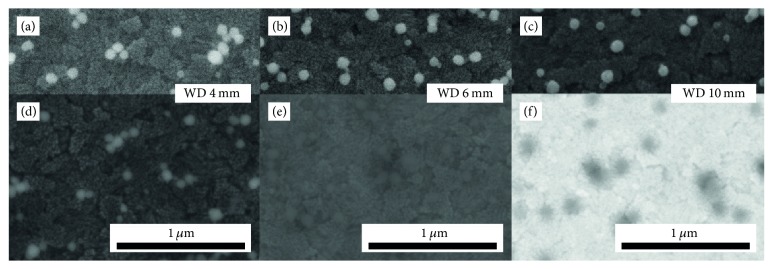
5 keV SE (a–c) and BSE (d–f) images of SiO_2_ NPs on ITO160 as a function of the WD given in the images. The image contrast was postprocessed for optimum visibility.

Figure 3 illustrates the dependence of the NP contrast on the WD at 5 keV on the* ITO160* substrate. SE contrast in Figures 3(a)–3(c) is only weakly affected by the WD whereas the BSE contrast (Figures 3(d)–3(f)) changes considerably. The NP contrast is positive for 4 mm WD (Figure 3(d)) which is unexpected considering the average atomic number of SiO_2_ NP ($\bar{\text{Z}}$ = 10) and the ITO layer ($\bar{\text{Z}}$ ≈ 28.5). The BSE contrast decreases to a very low value at approximately 6 mm WD (Figure 3(e)) and is inverted for further increasing WDs, for example, at 10 mm (Figure 3(f)). The NPs show a diffuse dark contrast under these conditions. Surprisingly SE and BSE contrast is similar at 4 mm WD (Figures 3(a) and 3(d)). The topography of the ITO layer (tile-like structures) can be clearly resolved in both images. This is an indication for a superposition of BSE material contrast and topography contrast, which will be discussed in detail later.

**Figure 4:**
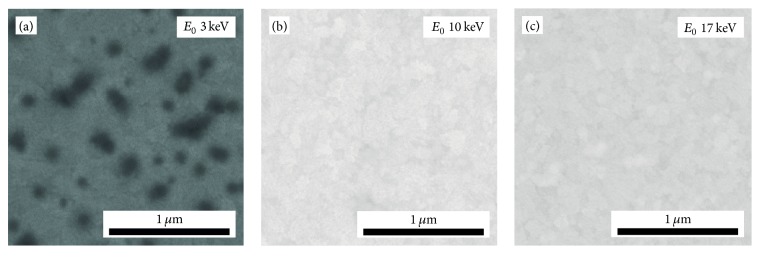
BSE images of NPs on ITO160 acquired at (a) *E*_0_ = 3 keV, (b) *E*_0_ = 10 keV, and (c) *E*_0_ = 17 keV at a constant WD of 10 mm. The image contrast was postprocessed for optimum visibility.

Figures 4(a)–4(c) show BSE images of SiO_2_ NPs on* ITO160* obtained with different *E*_0_ at 10 mm WD. The NP contrast is negative for 3 keV (Figure 4(a)). It is inverted if the PE energy exceeds 10 keV, where the contrast vanishes (Figure 4(b)). The contrast values remain negligible for further increasing *E*_0_ and NPs can be hardly recognized. Yet, digital image analysis yields a measurable contrast of 0.04 ± 0.01 for *E*_0_ = 17 keV (Figure 4(c)).

**Figure 5:**
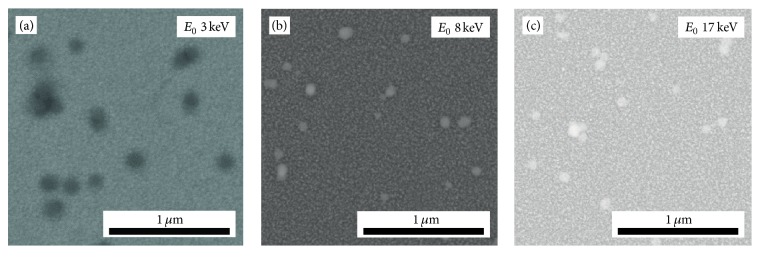
BSE images of NPs on ITO22 acquired at (a) *E*_0_ = 3 keV, (b) *E*_0_ = 8 keV, and (c) *E*_0_ = 17 keV at a constant WD of 10 mm. The image contrast was postprocessed for optimum visibility.

A PE energy dependent contrast inversion is also observed for the second substrate* ITO22*. This is illustrated in Figures 5(a)–5(c) which show BSE images of SiO_2_ NPs obtained at 10 mm WD with 3 keV, 8 keV, and 17 keV electrons. NP contrast is negative at the lowest PE energy of 3 keV (Figure 5(a)) and already clearly inverted at 8 keV (Figure 5(b)) indicating that the contrast inversion takes place between 3 and 8 keV. The contrast decreases slightly for the highest energy (Figure 5(c)). It is noted that the topography of the* ITO22* substrate shows smaller-scale features than* ITO160*, yet bright and dark areas can be distinguished indicating some roughness.

**Figure 6:**
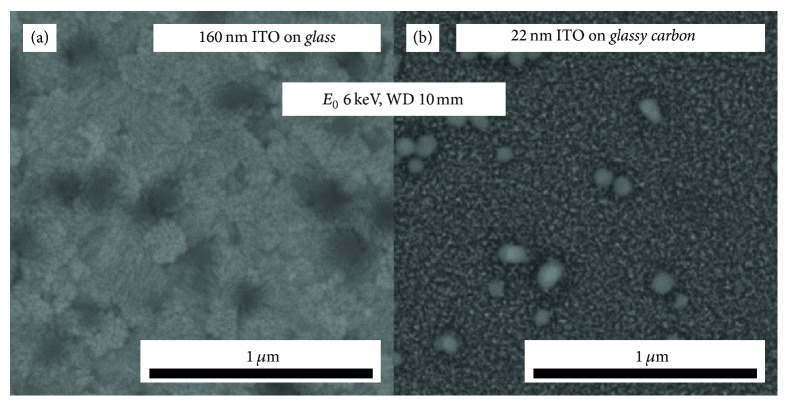
6 keV BSE images of (a) SiO_2_ NP on ITO160 and (b) SiO_2_ NP on ITO22 taken at WD = 10 mm. The image contrast was postprocessed for optimum visibility.

Another contrast inversion can be observed if BSE images of NPs on both substrates are directly compared. Figures 6(a) and 6(b) show 6 keV BSE images of NPs on* ITO160* (Figure 6(a)) and* ITO22* (Figure 6(b)) at 10 mm WD. Although identical imaging parameters were chosen, NP contrast is negative for* ITO160* and positive for* ITO22*. At first glance this indicates dependence on the substrate material, but it will be shown in the following that it is the result of the different ITO thicknesses.

A general characteristics of negative NP contrast are the diffuse NP appearance without any indication of the NP topography (Figures 3(e), 3(f), 4(a), 5(a), and 6(a)). Images of NPs with positive contrast show a substantially improved resolution.

## 4. Comparison of Measured NP Contrast with MC-Simulations

**Figure 7:**
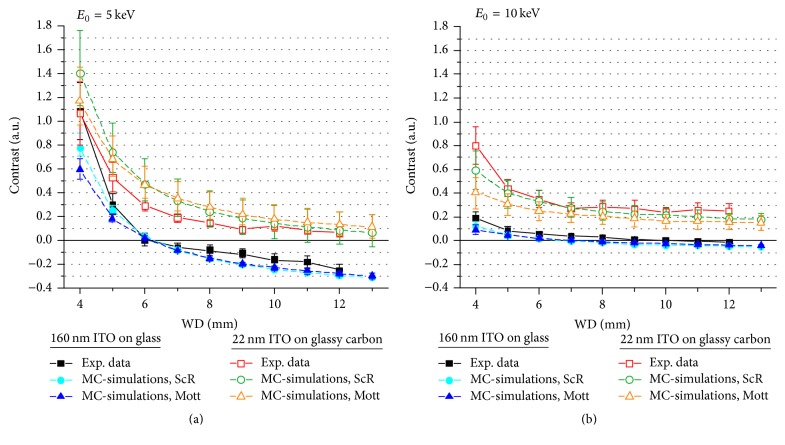
BSE contrast of SiO_2_ NP on ITO160 and ITO22 as a function of the WD. Comparison of experimental data (square symbols/solid lines) and MC-simulations using Screened Rutherford (circular symbols/dashed lines) and Cz Mott (triangular symbols/dashed lines) scattering cross-sections for (a) *E*_0_ = 5 keV and (b) *E*_0_ = 10 keV. Note the contrast inversion for ITO160 in (a) at a WD of 6 mm.

**Figure 8:**
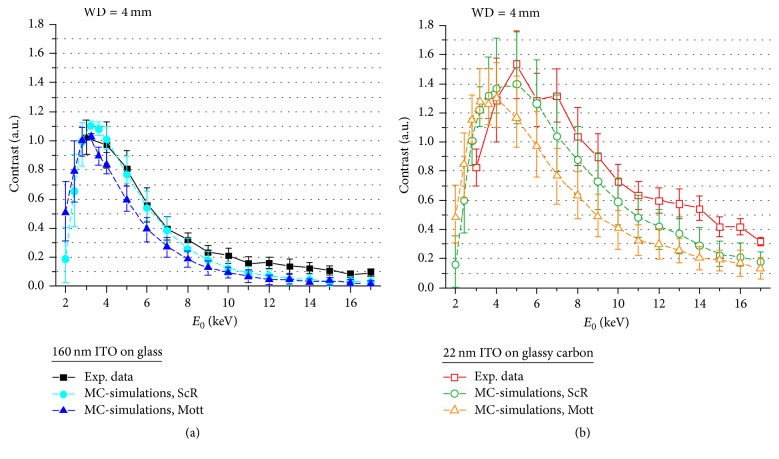
SiO_2_ NP BSE contrast as a function of *E*_0_ at 4 mm WD on (a) ITO160 and (b) ITO22. Experimental data are displayed by square symbols/solid lines, MC-simulations on the basis of Screened Rutherford are indicated by circular symbols/dashed lines and on the basis of Cz Mott cross-sections by triangular symbols/dashed lines.

**Figure 9:**
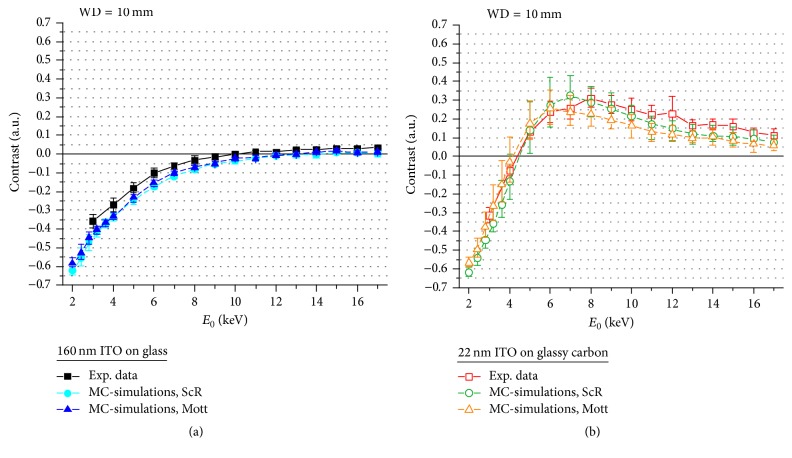
SiO_2_ NP BSE contrast as a function of *E*_0_ at 10 mm WD on (a) ITO160 and (b) ITO22. Experimental data are displayed by square symbols/solid lines; MC-simulations on the basis of Screened Rutherford are indicated by circular symbols/dashed lines and on the basis of Cz Mott cross-sections by triangular symbols/dashed lines.

Contrast inversions observed in Figures 3–6 suggest that simple interpretation of BSE images in terms of material contrast is not adequate for complex sample structures. In the following we will elaborate a systematic approach to understand and optimize NP contrast. Particularly the latter goal is motivated by the tedious and time-consuming trial and error procedure for a particular scenario. To understand BSE contrast formation we compare MC-simulations with experimental data in Figures 7–9. Square symbols and solid lines represent measured contrast values. Two different scattering cross-sections are used in the MC-simulation because the optimum choice of the scattering cross-section depends on the PE energy and the atomic number of the specimen materials. Circular symbols with dashed lines represent MC-simulations performed with ScR scattering cross-sections and triangular symbols with dashed lines indicate MC-simulations performed with Cz Mott cross-sections.

The errors of the MC-simulations for NP on* ITO22* are in general higher compared to NP on* ITO160*. This is related to the different thickness of the ITO layers, because all parameter variations have a much stronger impact on the contrast for* ITO22* than for the thicker ITO layer, especially the thickness variation by ±5 nm. This illustrates clearly the necessity of precise knowledge of simulation parameters for MC-simulations of complex nanoscaled structures.

Figure 7(a) shows the NP contrast as a function of the WD at a constant PE energy of 5 keV. It was already demonstrated in Figure 3 that NPs on* ITO160* undergo a contrast inversion from positive to negative values at approximately 6 mm WD (black square symbols and solid curve in Figure 7(a)). Analogous experiments on* ITO22 *(SEM images not displayed here) show that contrast values just decrease and approach zero for increasing WDs (red square symbols and solid curve in Figure 7(a)). MC-simulations confirm these observations and indicate that the NP contrast of* ITO22* might invert, too. The results for an increased *E*_0_ of 10 keV are presented in Figure 7(b). Like in the previous case, the NP contrast decreases for increasing WDs, but the overall NP contrast is smaller. The gradient of the curves is reduced for WDs > 5 mm, where the curves appear to asymptotically approach constant values. Contrast inversion for the* ITO22* substrate does not clearly occur, neither in the experimental data nor in the simulations. Contrast inversion for* ITO160* is only barely detectable in the experimental data and MC-simulations.

Figure 8 shows the NP contrast as a function of *E*_0_ at 4 mm WD for* ITO160* (Figure 8(a)) and* ITO22* (Figure 8(b)). Measurements (experimental images not shown here) and MC-simulations feature positive NP contrast with maxima at *E*_0_ ≤ 5 keV for both substrates and values approaching asymptotically zero for *E*_0_ > 5 keV. Contrast inversions do not occur in the experimentally accessible *E*_0_ range, but the MC-data indicates possible inversions at *E*_0_ ≤ 2 keV. However, this could not be verified due to the decreasing detector efficiency in the low-energy regime. MC-simulations for* ITO160* describe the energy dependence of the contrast well (Figure 8(a)). The match between measured and simulated contrast for* ITO22* is generally worse but still reasonable within the error bars (Figure 8(b)).

NP contrast is substantially different for WD = 10 mm (Figure 9) where the NP contrast is in general negative for small *E*_0_ compared to strong positive contrast for WD = 4 mm. The NP contrast for* ITO160* (Figure 9(a)) approaches zero with increasing *E*_0_ and inverts to small positive values. A contrast inversion is clearly observable for* ITO22* at 4 keV and maximum positive contrast occurs at 8 keV (Figure 9(b)). NP contrast is in general weaker compared to WD = 4 mm. Overall the calculated and measured data agree well and the contrast inversions shown in Figures 4 and 5 are confirmed by MC-simulations, although the inversion energies are slightly shifted for* ITO160* to approximately 10 keV compared to 13 keV in MC-simulations (Figure 9(a)).

With respect to the agreement of the MC-simulations and experimental data we note that a good agreement is generally obtained within the error bars. This underlines that MC-simulations are well suited to model BSE images of complex structures. The results of MC-simulations are less sensitive to the applied scattering cross-sections for the substrate with the thick ITO layer where only minor contrast changes are observed with a slightly better fit for Screened Rutherford cross-sections. Larger differences for the simulation results are observed for* ITO22* where simulations based on Screened Rutherford cross-sections clearly lead to a better agreement with the experimental data than Cz Mott cross-sections.

## 5. Discussion

The observed contrast inversions demonstrate the complex contrast formation in BSE imaging which prevents intuitive contrast interpretation in terms of material contrast. Positive NP contrast seems to contradict the expected negative material contrast between ITO ($\bar{\text{Z}}$ ≈ 24.5 for* ITO22* and 28.5 for* ITO160*) and SiO_2_ ($\bar{\text{Z}}$ = 10). In the following we discuss the origin of the anomalous BSE contrast formation using MC-simulations and derive guidelines to obtain optimum topography or material contrast.

**Figure 10:**
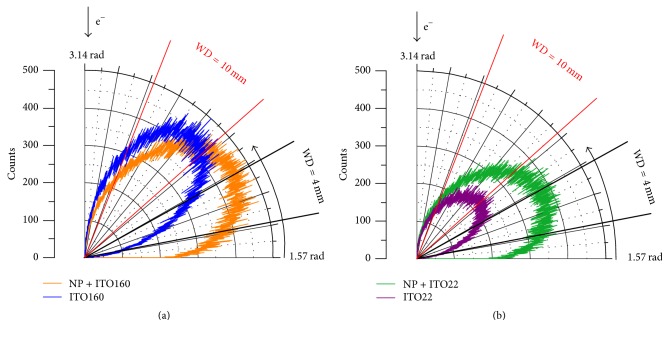
Polar diagrams of the angular BSE distribution at *E*_0_ = 5 keV for (a) NP + ITO160 (orange) and ITO160 (blue), as well as (b) NP + ITO22 (green) and ITO22 (purple). Straight lines enclose angular range of BSE detector at WD = 10 mm (red) and WD = 4 mm (black).

The WD-dependent contrast inversion (Figure 7(a)) can be explained by anisotropic angular scattering from the NP compared to the flat substrate. This is illustrated by Figure 10 where polar diagrams of simulated angular distributions of BSEs with 5 keV PE energy are presented.  Figure 10(a) shows the number of BSEs for 90 nm SiO_2_ NP on* ITO160* (orange) and without NP, that is, the mere substrate* ITO160* (blue). In Figure 10(b) the analogous data is shown for* ITO22* with NP (green) and without NP (purple). The straight red and black lines indicate the angular detection ranges at 4 mm and 10 mm WD. While the maxima of the distributions on the mere substrates lie between 2.25 rad and 2.35 rad, scattering of NPs on substrates is more concentrated in shallower angles (around 2.1 rad). Furthermore, the thicker ITO layer (blue in Figure 10(a)) shows a BSE contribution twice as high as the thinner one (purple in Figure 10(b)), because more PEs pass through the thin ITO layer without being backscattered. As a result, the BSE contribution of* ITO160* without NP exceeds the BSE contribution with NP at large WDs, for example, 10 mm, which is opposite to the behaviour at small WDs, for example, 4 mm (Figure 10(a)). This effect leads to the WD-dependent NP contrast inversion. In the case of the thin ITO layer (Figure 10(b)), BSE scattering is in general less intense compared to the BSE emission with NP which leads to positive NP contrast up to a WD of 12 mm.

**Figure 11:**
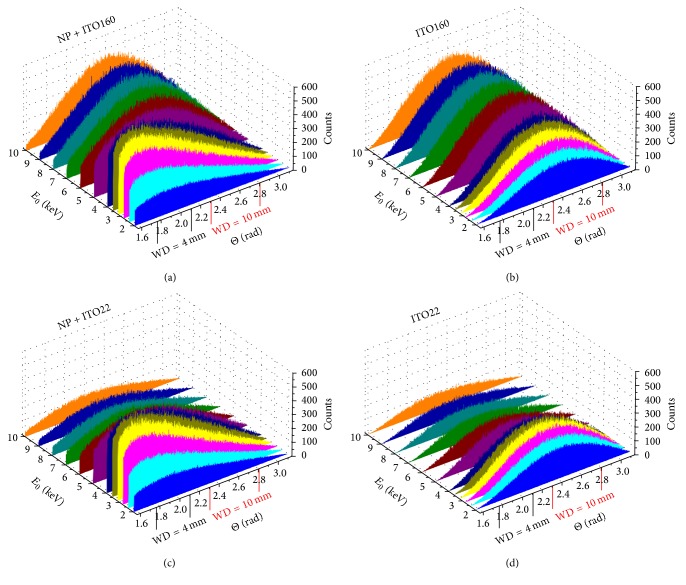
3D-plots of simulated angular BSE distributions as a function of the PE energy *E*_0_ for the 4 discussed cases: (a) NP on 160 nm ITO on glass (NP + ITO160), (b) 160 nm ITO on glass (ITO160), (c) NP on 22 nm ITO on glassy carbon (NP + ITO22), and (d) 22 nm ITO on glassy carbon (ITO22).

NP contrast as a function of the PE energy and PE energy dependent contrast inversions (Figures 8 and 9) can be understood by comparing simulated angular BSE distributions with and without NP on the two different substrates between 2 and 10 keV in Figure 11. A shift of BSE scattering towards smaller angles can be observed if a NP is present on the substrate (Figures 11(a) and 11(c)). The effect is enhanced with decreasing *E*_0_, because the probability of scattering inside the NP increases. The significant modification of the angular scattering range by the NPs is a direct result of the pronounced NP topography in combination with the topography information contained in low-angle BSEs as previously described by Robinson [15] and Joy [3]. This effect explains positive NP contrast at small WD in general. Maximum positive NP contrast is obtained at *E*_0_ between 3 and 4 keV (Figures 8(a) and 8(b)) where the shift of the BSE distribution towards smaller angles is most obvious (Figures 11(a) and 11(c)). Topography effects are less pronounced at larger WD which leads to the expected negative NP contrast for* ITO160 *up to ~10 keV (Figure 9(a)). Negative NP contrast for* ITO22* with the thin ITO layer is only found for small *E*_0_ (Figure 9(b)) where substrate backscattering is determined by the thin ITO layer (Figure 11(d)). Weak backscattering by the carbon substrate below becomes dominant for larger *E*_0_ which leads to NP contrast inversion at ~4 keV (Figure 9(b)). Negative NP contrast is generally enhanced with decreasing *E*_0_ (at least in the considered *E*_0_ range) due to the decreasing size of the interaction volume. The BSE intensity is then characteristic for the materials properties close to the sample surface, and NP contrast of* ITO160* and* ITO22* becomes similar. NP contrast generally approaches zero with increasing *E*_0_ (Figures 8 and 9) because the BSE intensity is dominated by the substrate properties. For *E*_0_ below 4 keV the overall intensity reduction is related to the decreasing detector efficiency for BSEs with energies below the threshold energy *E*_th_ = 3 keV.

From the preceding discussion we can derive recommendations regarding optimal conditions for BSE imaging of complex nanoscaled structures. If material contrast is desired, electron with large scattering angles must be preferentially detected to reduce topography effects. Hence, in the case of an annular semiconductor detector with limited angular detection range, large working distances (WD ≥ 10 mm) are required. PE energies should be small (*E*_0_ ≤ 4 keV for the samples considered in this work) to confine BSE emission to the region close to the sample surface. We did not explore extremely small *E*_0_ where the backscattering coefficient does not necessarily reflect the atomic number anymore [5]. We note that material contrast BSE images of NPs or any nanoscaled object on a bulk substrate will be diffuse even under optimum imaging conditions (Figure 5(a)). Strong positive NP contrast at small WDs (WD ≤ 4 mm) is always dominated by topography effects contained in low-angle BSE and leads to the best NP visibility. It is conceivable that the electron energy at maximum positive contrast depends on the size of the nanoscaled object which was already suggested in an early study [7] of the contrast of Au NPs on a Si substrate.

Finally, we add some remarks regarding the use of ScR and Cz Mott cross-sections in MC-simulations. Our results in the PE energy range of 3–17 keV show only small or moderate differences between calculated contrast values (Figures 8 and 9) with a tendency for convergence for *E*_0_ > 15 keV. These observations agree with statements by Shimizu and Ze-Jun [16] and Reimer [4], who report only minor differences between ScR and Cz Mott cross-sections if materials with small to intermediate *Z* values (like in this work) are considered, and PE energies exceed values of ~5 keV. Nevertheless, MC-simulations with ScR cross-sections generally yield a better fit with experimental data. This can be qualitatively understood because large-angle scattering in BSE SEM imaging is dominated by the central nucleus which is implied in Rutherford scattering. Therefore we recommend ScR cross-sections for MC-simulations of BSE contrast if experimental conditions are comparable to the conditions in our work.

## 6. Conclusions

The aim of this study was the understanding and optimization of backscattered electron (BSE) SEM contrast of SiO_2_ NPs on complex bulk substrates as a function of the primary electron energy *E*_0_ and working distance WD. Specifically SiO_2_ nanoparticles with 90 nm diameter on ITO/glass and ITO/glassy carbon substrates were studied. An annular semiconductor BSE detector was used in this work. The experimental NP contrast was quantitatively compared with results from MC-simulations which are well suited to model BSE SEM contrast. Our study allows deriving the following conclusions:

- BSE SEM images of NPs have to be interpreted with care in terms of material contrast because BSE images show NP contrast inversions depending on *E*_0_ and WD.
- Material-sensitive contrast is obtained for large scattering angles which are preferentially collected by using large WDs (WD ≥ 10 mm). Small *E*_0_ should be used to confine backscattering to regions close to the specimen surface. However, *E*_0_ values that are too small must be avoided because the detection efficiency of semiconductor BSE detectors decreases below the threshold energy *E*_th_. Topography contrast dominates for small WDs where a high fraction of BSEs with smaller scattering angles are collected.
- MC-simulations quantitatively agree with the experimental BSE contrast of NPs. It is mandatory to take the detection characteristics of the semiconductor BSE detector into account. The detector efficiency was assumed to linearly decrease with the BSE energy below the so-called threshold energy *E*_th_ which was assumed to be 3 keV in our case. Another prerequisite is that the material properties (composition, average atomic number, and material density) must be precisely known. This applies in particular to nanoscaled objects where discrepancies between real properties and assumed input parameters for MC-simulations lead to large errors.
- The scattering behaviour for primary electron energies used in this work (3–17 keV) and materials with small to intermediate *Z* values is best described by Screened Rutherford scattering cross-sections.

Material contrast BSE imaging of nanoscaled objects will profit from BSE detectors with lower *E*_th_ and improved resolution at low *E*_0_. A limitation of the intuitive interpretation of material contrast BSE imaging at *E*_0_ ≤ 1 keV is given by the behaviour of the backscattering coefficients which increase for low-*Z* materials and decrease for material with higher *Z* [5]. Moreover, scattering cross-sections for MC-simulations are missing for most atoms at these low electron energies.

## Figures and Tables

**Table 1:** Material parameters for NP and substrates used in MC simulations.

	Silica (NP)	ITO (160 nm)	ITO (22 nm)	Glass substrate	Glassy carbon substrate
Average atomic number $\overset{-}{\text{Z}}$	10	28.5	24.5	10	6
Average atomic weight	20	65.6	55.7	20	12
Material density (g/cm^3^)	2.2	7.1	5.0	2.2	1.3
Stoichiometry	SiO_2_	In_10_SnO_11_	In_7_SnO_12_	SiO_2_	C
